# Targeting p53 via JNK Pathway: A Novel Role of RITA for Apoptotic Signaling in Multiple Myeloma

**DOI:** 10.1371/journal.pone.0030215

**Published:** 2012-01-20

**Authors:** Manujendra N. Saha, Hua Jiang, Yijun Yang, Xiaoyun Zhu, Xiaoming Wang, Aaron D. Schimmer, Lugui Qiu, Hong Chang

**Affiliations:** 1 Division of Molecular and Cellular Biology, Toronto General Hospital Research Institute, Toronto, Ontario, Canada; 2 Department of Laboratory Medicine & Pathobiology, University of Toronto, Toronto, Ontario, Canada; 3 Department of Hematology/Oncology, Shanghai Children's Medical Center, Shanghai Jiaotong University, Shanghai, China; 4 Princess Margaret Hospital, Ontario Cancer Institute, Toronto, Ontario, Canada; 5 Institute of Hematology and Blood Disease Hospital, Chinese Academy of Medical Sciences and Peking Union Medical College, Tianjin, China; 6 Department of Laboratory Hematology and Medical Oncology, University Health Network, Toronto, Ontario, Canada; Penn State Hershey Cancer Institute, United States of America

## Abstract

The low frequency of p53 alterations e.g., mutations/deletions (∼10%) in multiple myeloma (MM) makes this tumor type an ideal candidate for p53-targeted therapies. RITA is a small molecule which can induce apoptosis in tumor cells by activating the p53 pathway. We previously showed that RITA strongly activates p53 while selectively inhibiting growth of MM cells without inducing genotoxicity, indicating its potential as a drug lead for p53-targeted therapy in MM. However, the molecular mechanisms underlying the pro-apoptotic effect of RITA are largely undefined. Gene expression analysis by microarray identified a significant number of differentially expressed genes associated with stress response including c-Jun N-terminal kinase (JNK) signaling pathway. By Western blot analysis we further confirmed that RITA induced activation of p53 in conjunction with up-regulation of phosphorylated ASK-1, MKK-4 and c-Jun. These results suggest that RITA induced the activation of JNK signaling. Chromatin immunoprecipitation (ChIP) analysis showed that activated c-Jun binds to the activator protein-1 (AP-1) binding site of the p53 promoter region. Disruption of the JNK signal pathway by small interfering RNA (siRNA) against JNK or JNK specific inhibitor, SP-600125 inhibited the activation of p53 and attenuated apoptosis induced by RITA in myeloma cells carrying wild type p53. On the other hand, p53 transcriptional inhibitor, PFT-α or p53 siRNA not only inhibited the activation of p53 transcriptional targets but also blocked the activation of c-Jun suggesting the presence of a positive feedback loop between p53 and JNK. In addition, RITA in combination with dexamethasone, known as a JNK activator, displays synergistic cytotoxic responses in MM cell lines and patient samples. Our study unveils a previously undescribed mechanism of RITA-induced p53-mediated apoptosis through JNK signaling pathway and provides the rationale for combination of p53 activating drugs with JNK activators in the treatment of MM.

## Introduction

Although mutations in the p53 gene occur in half of all cancers, approximately 90% of multiple myeloma (MM) cells retain a functional wild type p53 [1]–[3]. The low frequency of p53 alterations (mutations/deletions) in MM makes this tumor type an ideal candidate for p53-targeted therapies. Even in cancers retaining wild-type p53, p53 function is effectively inhibited which is primarily performed by the MDM2. Studies using small molecule inhibitors of the p53-MDM2 interaction such as nutlin and RITA (Reactivation of p53 and induction of tumor cell apoptosis) have shown the potential for pharmacological activation of p53 by disrupting the p53-MDM2 interaction as a new and promising anticancer strategy [4]–[8].

We have previously demonstrated an anti-myeloma activity of RITA mediated by activation of the p53 pathway [9]. RITA-induced apoptosis was shown to be associated with up-regulation of p53 and a pro-apoptotic target Noxa and down-regulation of p21 and MDM2 and an anti-apoptotic target Mcl-1. In addition, apoptosis was predominantly followed by extrinsic pathways [9]. Based on the previous reports on the apoptotic effect of RITA on different types of solid tumors, RITA-induced apoptosis is thought to be mediated by inhibition of the p53-MDM2 interaction by binding of RITA with p53 [8], [10]–[12]. However, a recent study by Nuclear Magnetic Resonance (NMR) indicated that RITA does not block the p53-MDM2 interaction in vitro [13]. Thus, whether binding to p53 is the only mechanism by which RITA increases p53 activity in cells is a matter of debate. It is highly possible that that RITA-induced activation of the p53 pathway can also occur in the mechanisms independent of inhibition of the interaction between p53 and MDM2.

In non-stressed normally growing cells, p53 degradation is not only mediated by its negative regulator MDM2, but also through binding with inactive form of c-Jun NH_2_-terminal kinase (JNK), which is one of the mitogen activated protein kinases (MAPKs), also known as stress-activated protein kinase (SAPK) [14]. In response to stress, JNK is activated through induction of cascades of two major MAPK families: MAP3K including ASK1 and MAP2K including MKK4 [15]. JNK signaling involves sequential activation of MAP3K, MAP2K, and JNK, which eventually leads to phosphorylation of c-Jun [16]. c-Jun is the founding member of the activator protein-1 (AP-1) family of transcription factors which bind to AP-1 elements in their target genes [17]. Recent studies have shown that JNK can directly or indirectly modulate expression of p53 and its targets and can positively influence apoptotic cell death [14], [18], [19]. Since JNK in association with p53 plays an important role in p53 stability, activation of p53 by stress and damage stimuli often correlates with induction of JNK [20]. Reportedly, JNK activation is one of the crucial pathways for apoptosis induction by the leading anti-MM agents such as proteasome inhibitors or immunomodulatory drugs (IMiDs), or various new candidate agents for MM [21]–[24].

Although a variety of mechanisms has been proposed to explain the activation of the p53 pathway in tumor cells there is still lack of evidence for functional linkage between JNK signaling and p53. The activation of the p53 pathway by RITA and the association of JNK and p53 by other anti-MM agents led us suggest that activation of the p53 by RITA may be mediated by JNK signaling pathway. Here we provide the evidence that RITA-induced activation of p53 in MM cells is dependent on JNK signaling. Detailed insights into molecular signaling pathways involved in RITA-induced apoptotic cell death may prove useful in the development of p53 based therapeutic approaches and strategies for JNK-mediated tumor targeting.

## Materials and Methods

### Patient samples and cell lines

Myeloma samples were collected from newly diagnosed patients. This study received written approval from the University Health Network Research Ethics Board in accordance with the Declaration of Helsinki. Cultured MM cell lines (MM.1S, 8226 and 8226R5) were collected from different sources and maintained as previously described [9]. NCI-H929, HeLa, MCF-7, and OCI-AML-3 cell lines were obtained from American Type Culture Collection (ATCC).

### Drug treatment

RITA and nutlin were purchased from Cayman Chemical and dissolved in dimethyl sulfoxide (DMSO) to create a 50 mM stock solution and stored at -20°C. Etoposide was purchased from Enzo Life Sciences (Farmingdale, NY). In each experiment, the final DMSO concentration was kept constant and did not exceed 0.05% (vol/vol). In some experiments, cells were simultaneously exposed to RITA and dexamethasone (DXM) (Cayman Chemical) (or 2-Cyano-3,12-dioxooleana-1,9-dien-28 oic Acid (CDDO) (REATA pharmaceuticals, TX). CDDO was prepared at 20 mM stock solutions in DMSO and was stored at -20°C. JNK-specific inhibitor, SP600125 and p53-transcriptional inhibitor, PFT-α were purchased from InvivoGen (San Diego, CA) and Enzo Life Sciences, respectively. After drug treatment, cells were harvested and subjected to further analysis as described below.

### Cell viability and apoptosis assays

Cell viability was assayed by MTT assay performed in triplicate at least twice as previously described [9]. To examine apoptotic cell death, MM cells were treated with various concentrations of RITA in the absence or presence of a SP600125 or PFT-α and stained for analysis by Flow cytometry with Annexin V-FITC (Abcam, MA) and propidium iodide (Sigma-Aldrich, St. Louis, MO). Data were analyzed using FlowJo software as described previously [9].

### Gene expression analysis and Quantitative Real Time PCR (qRT-PCR)

Total RNA was isolated using TRIzol reagent (Invitrogen, Carlsbad, CA) and the gene expression profile was evaluated using Illumina RNA analysis Beadchips (Illumina Inc. San Diego, CA) representing ∼48,000 human genes (Human HT12) as described earlier [9]. Expression of key genes in RITA-induced MM.1S cells involved in cell proliferation, cell-cycle arrest or apoptosis was analysed. To quantify and validate the expression of p53 target genes of interest at their mRNA level, qRT-PCR assays using glyceraldehyde-3-phosphate dehydrogenase (GAPDH) as a reference gene were performed as described previously [9].

### Immunoblotting

Western blot analysis of the whole cell lysates obtained from the cells treated with RITA in the absence or presence of the inhibitors or siRNAs were performed as described previously [9]. Primary antibodies were from the following manufacturers: Santa Cruz Biotechnology (Santa Cruz, CA): p53 (DO-7) and β-actin; Abcam: NOXA; Cell Signaling Technology (Danvers, MA): Mcl-1, JNK1/2, caspase-3 and PARP; Signalway Antibody (Pearland, TX): ASK-1-p, MKK4-p, c-Jun, c-Jun-p and 4E-BP1; Biolegend (San Diego, CA): α tubutlin. Goat anti-mouse and anti-rabbit secondary antibodies conjugated to horseradish peroxidase were purchased from Cell Signaling and Santa Cruz Biotechnology, respectively.

### Genetic Knockdown of selective target genes

H929 or MM.1S cells were transfected with target specific siRNAs for JNK (Sigma) or p53 (Invitrogen) or control scrambled siRNA (Invitrogen) using the Cell Line Solution Kit V (Amaxa, GmbH, Cologne, Germany) according to the manufacturer's instruction with the Amaxa Nucleofector II device (Amaxa). Custom siRNA sequence for JNK simultaneously targets JNK1 and JNK2 [25]. Following transfection, cells were treated with RITA and analysed for inhibition of activation of the p53 and apoptotic targets including caspase-3 and PARP. The effect of cell viability and apoptosis induction by RITA following the knockdown of JNK or p53 was analysed by MTT assay and FCM, respectively.

### Chromatin immunoprecipitation (ChIP) assays

ChIP assays were performed in MM.1S and H929 cells treated with RITA or DMSO control as described by Chen et al [26]. In brief, formaldehyde cross-linked chromatin was isolated from 5×10^7^ cells followed by IP with phosphorylated c-Jun antibody or normal rabbit immunoglobulin (IgG) bound to ChIP grade sephadex A Bead (Cell Signaling) according to the manufacturer's instruction. DNA was eluted from the beads and reverse cross-linked according to the protocol. Polymerase chain reaction was used to analyze the immunoprecipitated DNA with the use of primers against AP-1 binding site of p53 promoter region (fwd-AGGAGCCTCGCAGGGGTTGAT, rev-CCAATCCAGGGAGTGTCACCG) or GAPDH (fwd-ACATCGCTCAGACACCATG, rev-TGTAGTTGAGGTCAATGAAGGG).

### Statistical analysis

The synergistic effect [combination index (CI) <1.0] of the combination of RITA and DXM or CDDO was analyzed using CalcuSyn (Biosoft, Cambridge, UK), a software program based on the Chou-Talalay method, as described previously [7], [9]. An isobologram is a graph that indicates affected fraction and CI. Statistical significance levels were determined by two-tailed *t* test analysis. *p* values of <0.05 were considered significant.

## Results

### Gene expression profiling (GEP) by microarray identified differentially expressed genes in JNK signaling pathway

**Figure 1:**
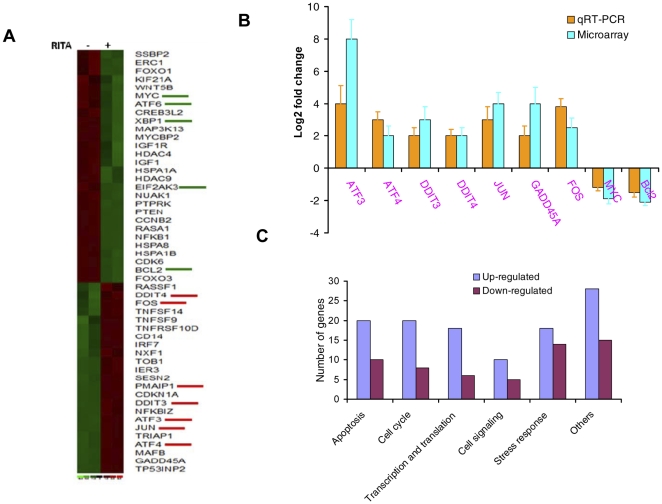
RITA induces transcriptional regulation of a set of p53 target genes. Gene expression analysis by microarray in MM.1S cells after 6 hrs treatment with 1 µM RITA. (A) Shown is the heat map of genes differentially expressed in MM.1S cells. Vertical rows indicate separate arrays, and horizontal rows indicate genes. A number of differentially expressed genes associated with stress responses are labeled with green and red lines. Green indicates low expression; red indicates high expression. (B) mRNA levels of the genes were detected by qRT-PCR in MM.1S cells 6 hrs after treatment with 1 µM RITA. Values were normalized to those obtained for GAPDH and expressed as log2 fold induction over DMSO treated cells (mean ± SD). (C) Genes are categorized according to their functions and represented in the figure.

Our GEP by microarray data of MM.1S cells treated with RITA demonstrates transcriptional triggering of apoptotic cascades, down-regulation of growth/survival kinases, up-regulation of unfolded protein responses (UPR), and induction of stress kinases. A total of 51 selected genes differentially expressed between RITA-treated and DMSO control treated MM.1S cells are represented in the heat map (Figure 1A).

To confirm the results of the gene expression by microarray, qRT-PCR validation was performed on the RNA samples used for the initial array. A full list of the validated primers can be found in the Table S1. The expressions of the genes in RITA-induced MM.1S cells by qRT-PCR (Figure 1B), were observed to have consistent dysregulation between RITA-treated and control DMSO-treated cells and were similar to those changes seen by microarray analysis. Of note, ∼2–4 fold increase in the stress responsive genes, ATF3, ATF4, DDIT3, DDIT4, c-Jun and FOS, was observed upon RITA-stimulation (Figure 1A, B). Consistent with the p53 cellular functions, we found that 62 of the 229 genes in RITA-induced MM.1S cells were involved in apoptosis, cell cycle regulation, cell growth and differentiation, DNA repair and chromatin modification, or transcription regulation. Importantly, a significant number of genes (∼46) were associated with different types of stress signaling including p53 and JNK signaling (Figure 1C, Table S2). Of greatest interest from the microarray analyses was the ∼3-fold up-regulation of c-Jun, one of the substrates of JNK. These results indicated that JNK-mediated signaling is involved in RITA-induced cell death in MM cells. We subsequently focused our analysis on the activation of c-Jun/JNK signaling.

To identify the most relevant biologic mechanisms, pathways, and functional categories of the (229) genes affected by induction of c-Jun, we used Ingenuity Pathways Analysis (IPA) software (Ingenuity Systems, Redwood City, CA). Using IPA with false discovery rate (FDR) of 10% and fold change cut-off of ±2, we evaluated the interaction and functional importance of the signaling pathways involving genes significantly dysregulated in MM.1S cells treated with RITA or DMSO control. IPA analysis of the 120 genes differentially expressed between RITA treated and non-treated MM.1S cells revealed two significant networks which target the JNK pathway (Figure S1). The two networks represent the proteins associated with cell signaling, cellular growth and proliferation, cell cycle, cellular development and JNK signaling pathways. Molecules associated within these pathways are listed in Table S2.

### RITA induces activation of JNK in MM cells

**Figure 2:**
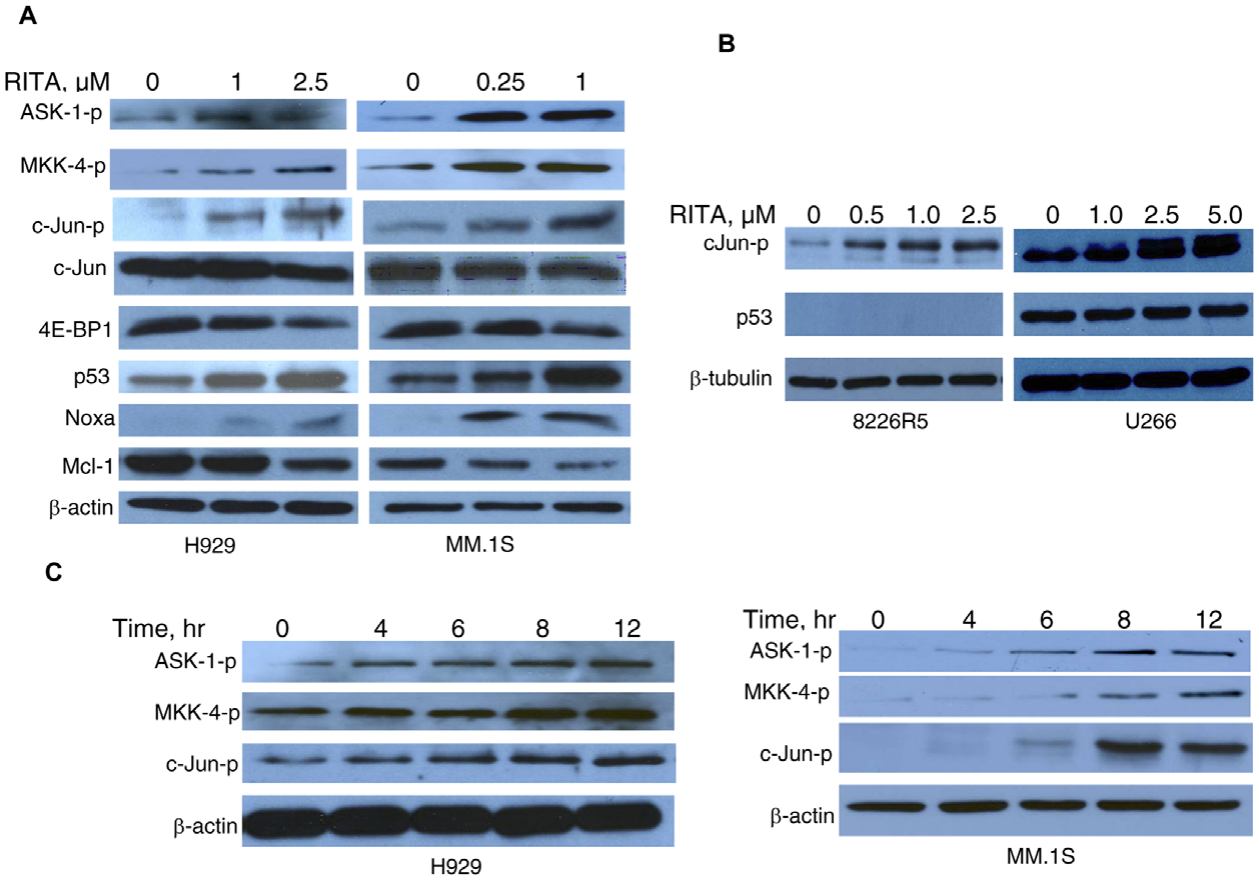
RITA induces activation of JNK signaling in MM cells. (A) H929 and MM.1S cells (harboring wild type p53), and (B) 8226R5 (p53 null) and U266 (p53 mutant) cell lines were treated with different concentrations of RITA for 8 hrs. (C) H929 or MM.1S cells were treated with 2.5 µM or 1 µM RITA, respectively for different time periods (4–12 hrs). The cells were then harvested and subjected to preparation of whole-cell protein lysates for detection of the indicated proteins by Western blot analysis.

JNK is responsible for the phosphorylation of a variety of proteins including downstream kinases and transcription factors such as c-Jun with subsequent transcriptional AP-1 activation [27]. Indeed, c-Jun phosphorylation is widely regarded as an inevitable consequence of JNK activation. MM cell lines of different p53 status were treated with RITA and c-Jun amino terminal phosphorylation was examined by immunoblotting using a phospho-specific (Ser73) c-Jun antibody (Figure 2). We found that treatment of myeloma cells (H929 and MM.1S) with RITA resulted in a dose-dependent increase in the phosphorylation of c-Jun. However, the protein level of total c-Jun remained relatively constant during the course of treatment (Figure 2A). Based on this data, we then attempted to identify the upstream signaling molecules involved in the activation of JNK in cells treated with RITA. Western blot analysis revealed that H929 or MM.1S cells treated with RITA for 8 hrs induced phosphorylation of ASK-1 (Ser966) and MKK-4 (Ser80), representative members of MAP3K and MAP2K family, respectively. These events were followed by up-regulation of p53, and a pro-apoptotic protein, Noxa; down-regulation of Mcl-1, an anti-apoptotic protein, and 4E-BP1, a survival factor in JNK pathways (Figure 2A). We compared the effect of RITA on c-Jun activation in the wild type p53 expressing H929 and MM.1S cells with that in the 8226R5 p53 null and mutant p53 expressing U266 cells. Interestingly, the activation of c-Jun induced by RITA was found to be p53-independent, i.e., up-regulation of phosphorylated c-Jun was not only observed in MM cells harboring wild type p53 but also in cells harboring null or mutant p53 (Figure 2B). However, as described in our previous report, RITA induced apoptosis only in cells harboring wild type p53 [9]. Kinetic analysis showed that RITA treatment induced phosphorylated c-Jun level in H929 and MM.1S cells in a time-dependent manner. Phosphorylation of ASK-1 and MKK4 was also observed at the similar fashion (Figure 2C). These results are in line with our previous study in which time-dependent activation of p53 was observed in these two cells lines [9]. Taken together these results demonstrate that RITA-induced apoptosis in MM cells is mediated by activation of JNK signaling cascade.

### Effect of other nongenotoxic or genotoxic drugs on JNK activation in MM

**Figure 3:**
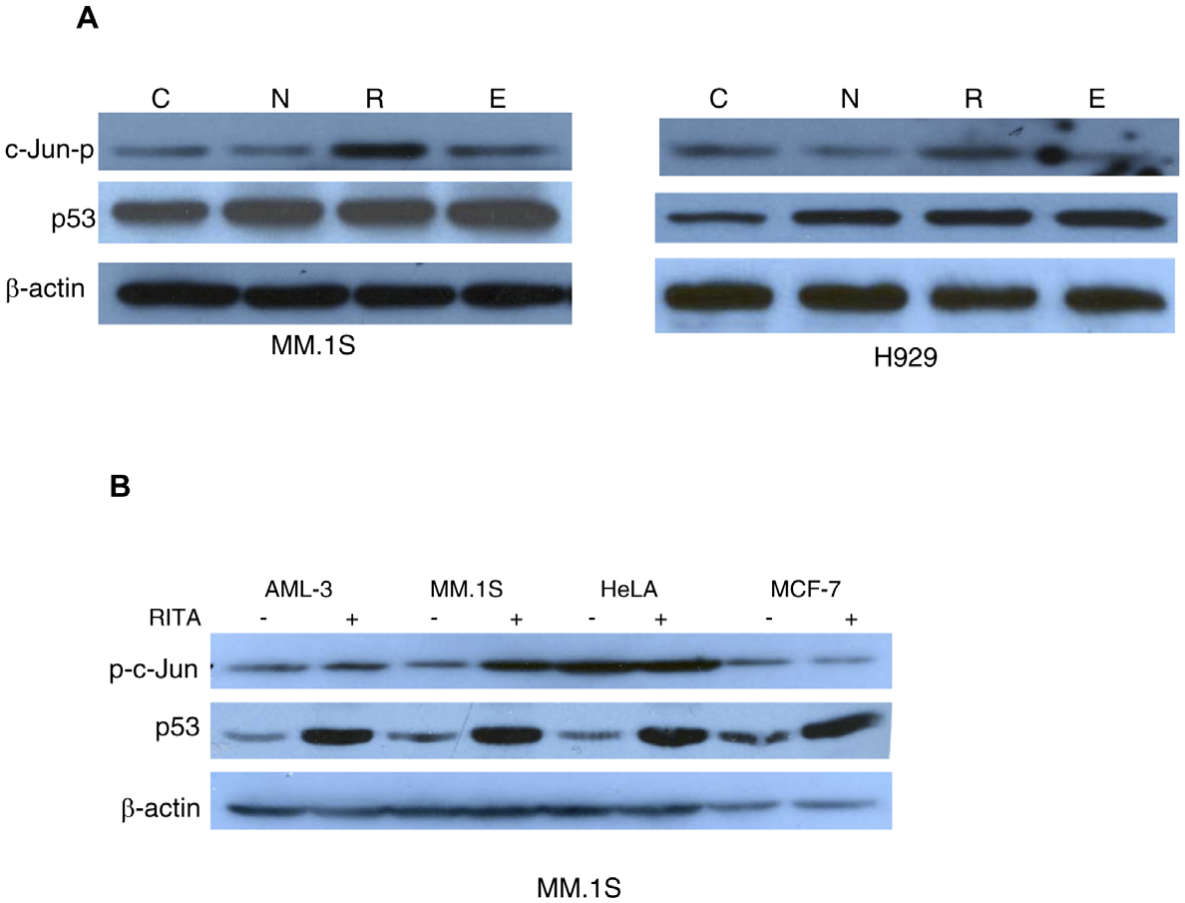
RITA-specific activation of JNK is selective to MM cells. (**A**) MM.1S or H929 cells were treated with RITA (1 µM for MM.1S or 2.5 µM for H929), nutlin (5 µM), or etoposide (5 µM) along with DMSO control. 8 hrs after treatment, cells were harvested for Western blot analysis and detected for the up-regulation of c-Jun-p and p53. C, N, R, and E indicate cells treated with DMSO control, nutlin, RITA and etoposide, respectively. (B) Three different types of cells: AML-3 (myeloid leukemia), HeLa (cervical cancer), and MCF-7 (breast cancer) were treated with 1 µM RITA or DMSO for 8 hrs. MM.1S cells were treated in a similar manner as controls. The cells were harvested for analysis by Western blot which were probed with the indicated antibodies.

Having shown that small molecule RITA induced activation of JNK in MM cells, we examined whether the activation of JNK is specific to RITA. MM.1S or H929 cells were treated with the nongenotoxic small molecules nutlin or RITA and a genotoxic agent etoposide and examined for activation of JNK. Western blot analysis of the samples harvested from MM cells treated with these agents revealed the phoshphorylation of c-Jun in cells treated with RITA. However, phosphorylation of c-Jun was not significantly modulated when the cells were treated with nutlin or etoposide. These results suggest that activation of JNK in MM cells is RITA specific (Figure 3A).

### Effect of JNK activation induced by RITA in other cancer cell types

Since RITA induced JNK activation in MM cells, we next attempted to see whether RITA-induced activation of JNK can be observed in other types of cancer cells. We evaluated the effect of RITA on JNK activation in additional 3 different types of cell lines harboring wild type p53, e.g., AML-3 (myeloid leukemia); HeLa (cervical cancer); and MCF-7 (breast cancer). The activation of p53 induced by RITA has been reported in HeLa [28] and MCF-7 cell lines [12]. MM.1S cell line was used as a control for RITA treatment. All cells were treated with 1 µM RITA for 8 hrs. Although activation of p53 was found in all of the cell lines upon RITA treatment, RITA-induced phosphorylation of c-Jun was observed in MM.1S cells but phosphorylation level of c-Jun was not significantly changed in other type of cells. These results suggest that RITA-induced activation of JNK is likely specific to myeloma cells (Figure 3B).

### JNK specific inhibitor or JNK siRNA inhibited the activation of p53 and p53-mediated apoptosis

**Figure 4:**
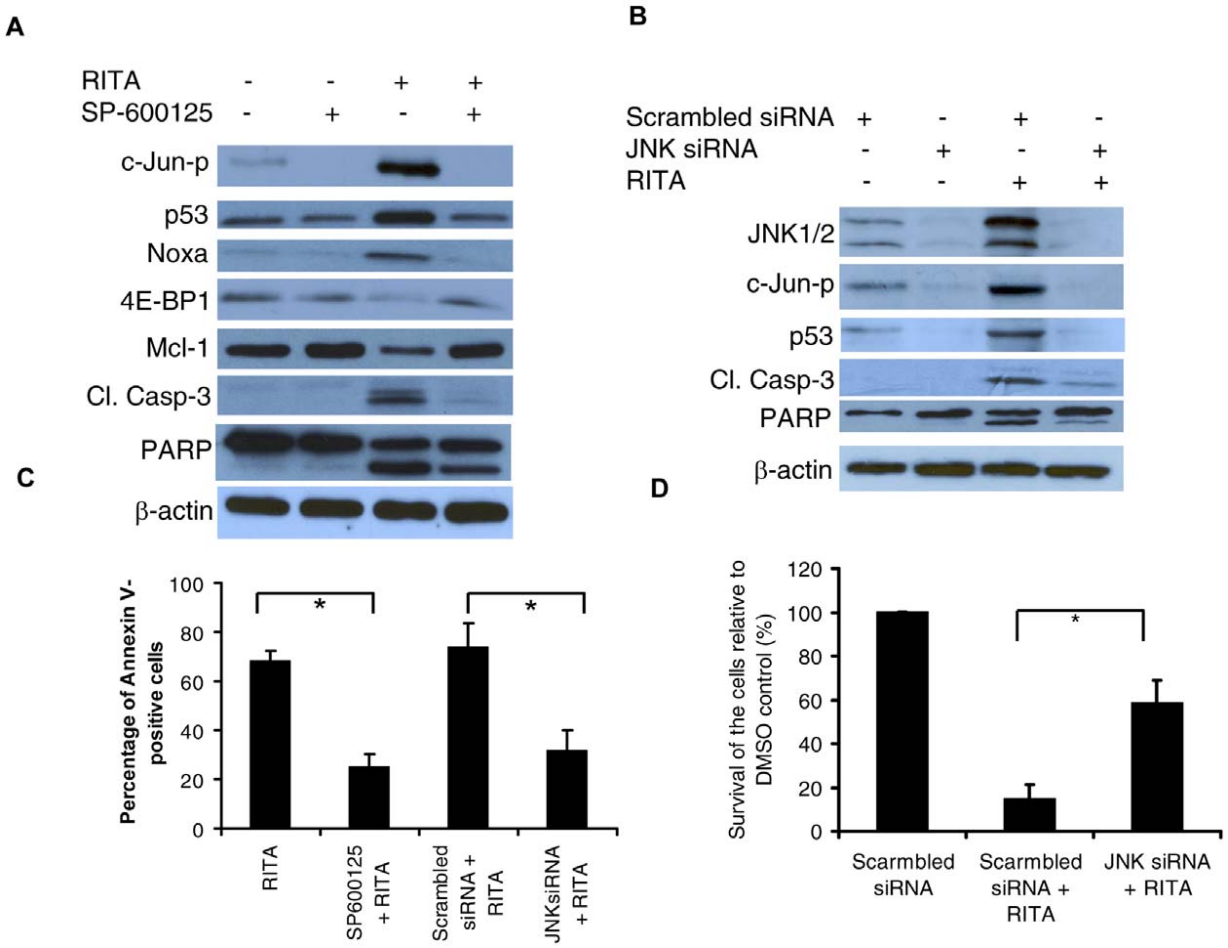
JNK activation is required for RITA-induced apoptosis. H929 cells were pretreated with SP600125 for 30 min (A) or transfected with scrambled or JNK siRNA for 24 hrs (B) and then treated with RITA for additional 8 hrs. The cells were then harvested and subjected to preparation of whole-cell protein lysates for detection of the indicated proteins by Western blot analysis. Cleaved caspase-3 is indicated as cl. caspase-3. (C) H929 cells pre-treated with SP600125 or transfected with JNK siRNA were further treated with 2.5 µM RITA for 48 hrs. RITA treated H929 cells were analysed by FCM for Annexin-V binding to measure the apoptosis. (D) Silencing of JNK suppresses RITA-induced cytotoxicity in MM cells. H929 cells transfected with either JNK siRNA or negative-scrambled siRNA and 48 hours later cells were treated with RITA (2.5 µM) or DMSO. 48 hrs treatments, cell viability were measured by MTT assay. *p <0.05 versus the corresponding cells treated with RITA in the absence of SP600125 or transfected with scrambled siRNA (Student's *t* test).

In order to clarify the involvement of JNK, we first investigated the role of JNK in the regulation of p53-mediated apoptosis induced by RITA in MM cells by using a JNK specific inhibitor, SP-600125 which exhibits significant selectivity for JNKs leading to inhibition of both phosphorylation of c-Jun and JNKs [29]. To this end, we treated H929 cells with RITA in the absence or presence of SP-600125 and analyzed the expression of the proteins associated with p53-mediated apoptosis (Figure 4). We found that, presence of SP600125 abrogated the ability of RITA to up-regulate phosphorylated c-Jun level. Concurrently, RITA-induced p53 activation was also inhibited by SP-600125. In addition, the up-regulation of Noxa, and down-regulation of 4E-BP1 and Mcl-1 induced by RITA also inhibited (Figure 4A). To further understand specific inhibition of JNK activation, JNK was selectively knocked down by siRNA approach. Similar to the results obtained by pharmacological inhibitor of JNK, activation of the phosphorylation of c-Jun as well as p53 was inhibited in JNK knocked down H929 cells treated with RITA (Figure 4B). Functionally, p53-dependent apoptosis of H929 cells was inhibited by both SP-600125 and JNK siRNA as evidenced by reduction of cleavage of caspase-3 and PARP by Western blot analysis (Figure 4A,B) and inhibition in Annexin V binding by FCM (Figure 4C). In addition, knocking down of JNK suppressed the growth inhibitory effect of RITA in H929 cells (Figure 4D). These results collectively indicate that activation of p53 induced by RITA is mediated by the activation of JNK and strongly suggest that JNK plays a critical role in mediating RITA-induced apoptosis.

### Chromatin immunoprecipitation assay (ChIP) revealed the binding of activated c-Jun to the p53 promoter region

**Figure 5:**
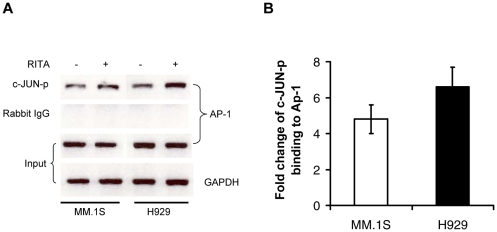
Activated c-Jun binds to AP-1 binding site of p53 promoter region. 8 hrs after exposure of MM.1S and H929 cells to 1 and 2.5 µM RITA, respectively, cells were prepared for ChIP with phosphorylated c-Jun, or control IgG antibodies. Cells treated with DMSO were used as controls. This was followed by PCR amplification of the target promoter sequence. (A) The normalized controls were performed by GAPDH for input DNA of MM.1S and H929 cell lines. (B) Experiments were repeated twice, and the average fold differences in relative enrichment (ChIP/IgG) over the GAPDH control is represented.

Having shown an important role of JNK signaling in p53 induction, we investigated whether RITA-induced activation of p53 is mediated by direct binding of c-Jun in the AP-1 binding site of the p53 promoter region. The p53 promoter contains a conserved AP-1-like element that differs from a consensus AP-1 site by a single base-pair exchange (termed PF-1 site) [30]. The binding of c-Jun to p53 promoter was studied by PCR using primers that flank AP1 site which amplify a 350 bp region. Phosphorylated c-Jun antibody immunoprecipitated an increased proportion of the region of the p53 promoter containing AP-1 site in both MM.1S and H929 cells treated with RITA, whereas the control antibody (IgG) failed to precipitate it (Figure 5A). Quantitative analysis showed a ∼5 and 7-fold increase of c-Jun binding to the p53 promoter in RITA-treated MM.1S and H929 cells, respectively, in comparison to DMSO control treated cells (Figure 5B). Our results clearly demonstrate that upon RITA stimulation phosphorylated c-Jun binds to p53 promoter for the induction of p53 transcriptional activity.

### Inhibition of p53 transactivation by p53 transcriptional inhibitor or p53 siRNA prevents activation of c-Jun

**Figure 6:**
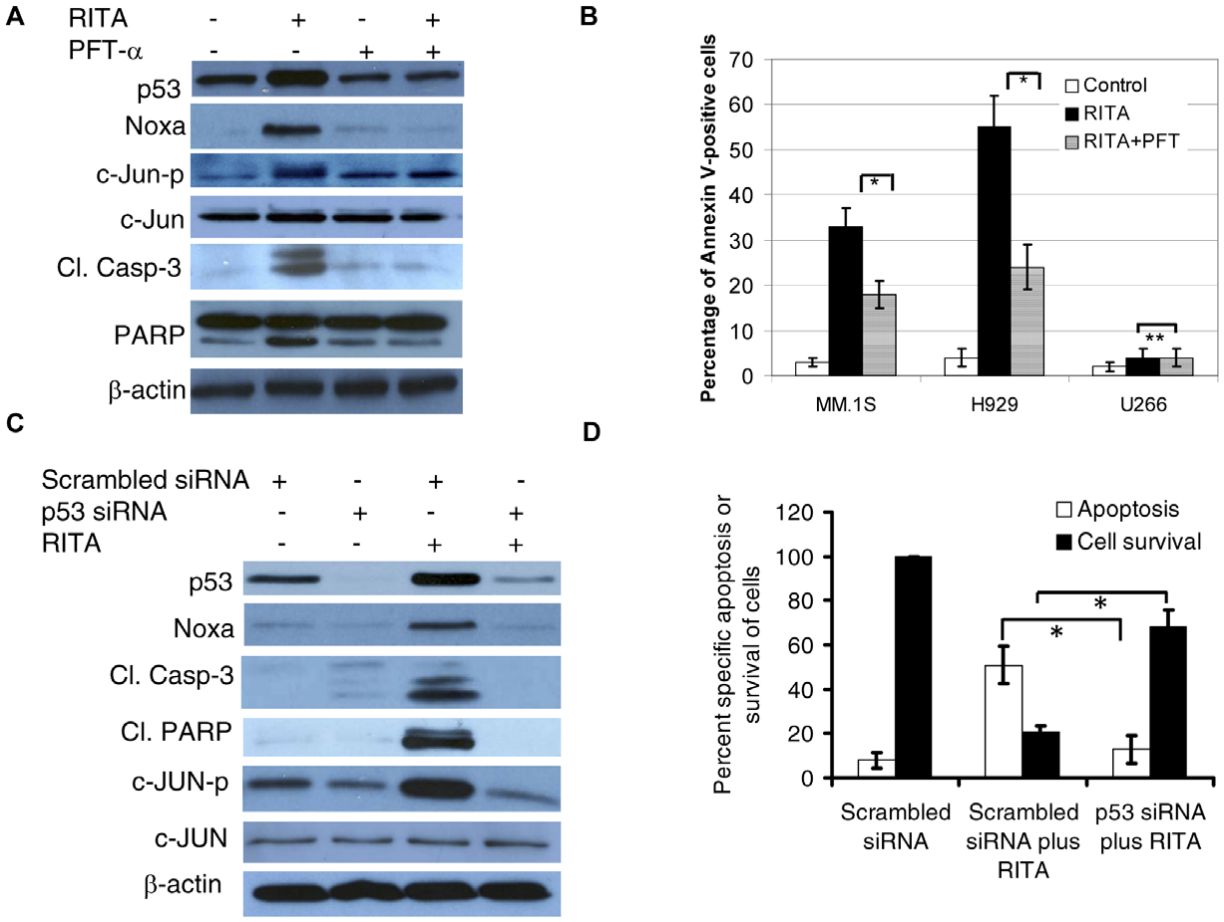
Blocking of p53 transactivation inhibits RITA–induced apoptosis. (A) Inhibition of p53 transcription inhibits activation of c-Jun in MM cells. H929 cells were pretreated with 15 µM PFT-α for 1 hr and then treated with 2.5 µM RITA for additional 8 hrs. The cells were then harvested and subjected to preparation of whole-cell lysates for detection of the indicated proteins by Western blot analysis. (B) p53 transcriptional inhibitor inhibits induction of apoptosis in MM cells harboring wild type p53. MM.1S, H929, and U266 cells were pretreated with PFT-α for 1 hr and then treated with RITA for additional 48 hrs. The cells were then analysed for Annexin V binding by FCM. *p<0.05 versus the corresponding cells treated with RITA in the absence of PFT-α (Student's *t* test), **statistically not significant. (C) and (D) Genetic Knockdown of p53 inhibits induction of apoptosis in MM cells. (C) MM.1S cells were exposed to 1 µM RITA for 8 hrs after transfection with scrambled or p53 siRNA for 48 hrs, cells were harvested for analysis by Western blot, and blots were probed with specific antibodies. (D) 24 hrs after transfection of MM.1S cells with scrambled or p53 siRNA, MM.1S cells were treated with RITA (1 µM) for additional 48 hrs, and apoptosis and cell viability was measured by FCM and MTT assay, respectively. *p<0.05 versus the corresponding RITA treated H929 cells transfected with scrambled siRNA (Student's *t* test).

Given the roles of JNK associated with induction of p53-mediated apoptosis in response to RITA, we next examined the role of p53 transcription by using a p53 transcriptional inhibitor, PFT-α, a specific inhibitor of p53 transcriptional targets. As shown in Figure 5A, PFT-α inhibited the up-regulation of p53 and Noxa as well as phosphorylation of c-Jun induced by RITA in H929 cells. Moreover, the apoptosis induction by RITA was also inhibited by PFT-α as evidenced by inhibition of cleavage of caspase-3 and PARP (Figure 6A) and inhibition of Annexin V binding in both MM.1S and H929 cells with wild-type p53 but not in U266 cells with mutant p53 (Figure 6B). These results suggest that p53 is involved in RITA-induced apoptosis of MM cells and confirm the linkage between p53 and JNK activation. To confirm the p53-dependent induction of apoptosis by RITA, using siRNA approach, we specifically knocked down p53 in MM.1S cells which was followed by assessment of p53 targets and its apoptotic effect. Silencing of p53 was associated with significant inhibition of RITA-induced activation of Noxa and cleavage of caspase-3 and PARP (Figure 6C). Importantly, similar to the results obtained from the inhibition of p53 transcription by PFT-α, RITA-induced phosphorylation of c-Jun was inhibited when p53 expression was silenced by siRNA. These results suggest the establishment of a positive feedback loop between p53 and JNK. In addition, knockdown p53 expression attenuated the RITA-induced increase of Annexin V-positive cells and inhibition of cell survival. For example, apoptosis induction by RITA in MM.1S cells was reduced from 52% to 15% in RITA-induced H929 cells transfected with p53 siRNA. Similarly, silencing p53 in MM.1S cells prevented the killing of cells mediated by RITA (Figure 6D). These results further confirm that RITA-induced apoptosis in MM cells is p53-dependent.

### RITA in combination with other JNK activating drugs displays synergistic cytotoxic responses

**Figure 7:**
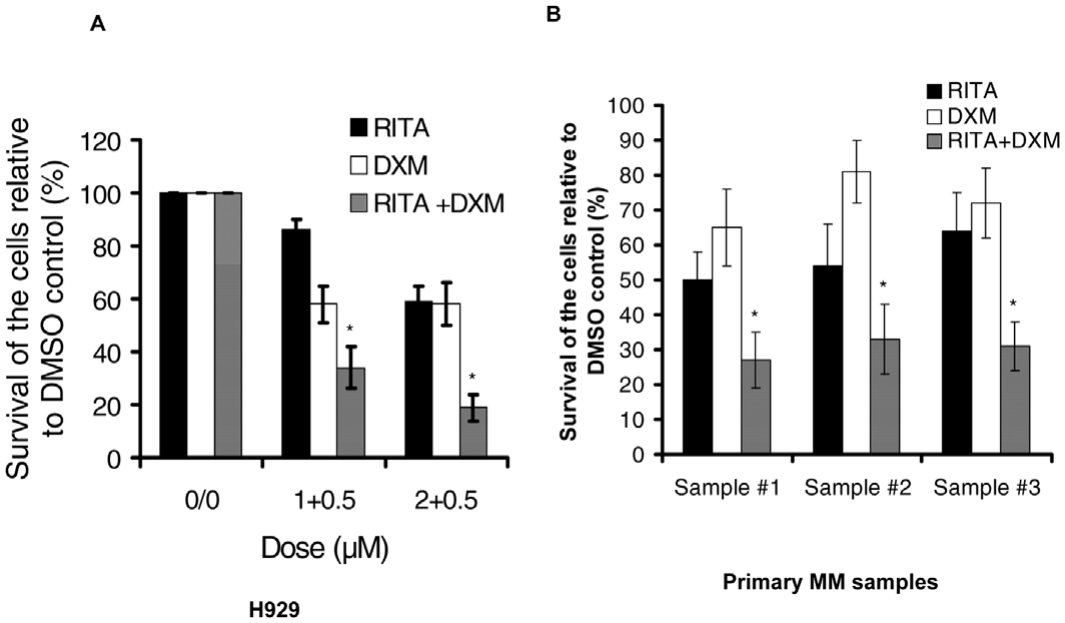
Combination RITA and dexamethasone induces synergistic cytotoxicity in MM cell lines and primary MM samples. (A) H929 cells were treated with 1 and 2 µM RITA, in combination with 0.5 µM DXM. (B) Freshly isolated primary MM samples were for treated with 5 µM RITA and 1 µM DXM. 48 hrs treatments of the cells, the viability were measured using MTT cell viability assays. Data are mean ±SD of triplicate samples. *p<0.05 versus the samples treated with RITA or DXM alone (Student's *t* test).

Having shown that RITA induces apoptosis via activation of the JNK signaling pathway, we further examined the combined cytotoxic effect of RITA and DXM, a conventional chemotherapeutic as well as an activator of JNK [31]. The effects of combination of RITA and DXM were assessed on the viability of MM cell lines and primary MM samples. We examined possible additive or synergistic anti-proliferative effects of RITA and DXM following 48 hours of treatment of H929 cells with lower doses (1 and 2 µM) of RITA combined with 0.5 µM DXM. Treatment of H929 cells with RITA or DXM alone induced only 10 to 40% cell killing which was synergistically enhanced to 65% (CI, 0.88) and 80% (CI, 0.74), respectively (Figure 7A) in RITA plus DXM combination (Table 1, Figure S2A). We next confirmed the cytotoxic response of RITA in combination with DXM in MM patient samples. The combination of 5 µM RITA and 1 µM DXM induced a synergistic cytotoxicity (CI = 0.54−0.72) in 3 primary MM samples (Table 2, Figure S2B). The synergistic anti-myeloma activity of the two agents was clearly demonstrated by a leftward shift of the dose response curve as well as isobologram and CI analyses in both H929 cell lines and primary MM samples (Figure S2A,B). To further understand the clinical significance of JNK activation in RITA-induced apoptosis we investigated the cytotoxic effect of RITA by combining it with CDDO, a known JNK activator [32]. First, dose responses of CDDO were examined in MM.1S and H929 cells after treating the cells with different concentrations of CDDO for 48 hrs. Results showed a dose-dependent killing of MM cells by CDDO (Figure S3A). Next, MM.1S or H929 cells were treated with low doses of RITA (0.25–1.0 µM) with a fixed dose of CDDO (0.5 µM) for 48 hrs and viability was measured. As shown in Figure S3B, in MM.1S cells the combination of 0.5 µM CDDO with either 0.25 or 0.5 µM RITA displayed a synergistic cytotoxic response with a CI value of 0.83 and 0.62, respectively. Similarly, combination of 0.5 µM CDDO with 0.5 or 1.0 µM RITA showed a synergistic cytotoxic response in H929 cells in which CI value was 0.92 and 0.87, respectively.

**Table 1:** Affected fractions and combination indices with RITA and DXM combination on H929 cell lines.

RITA (µM)	DXM (µM)	Affected fraction (FA)	Combination indices (CI)
1	0.5	0.66	0.88
2	0.5	0.81	0.74

Notes; A CI of less than, equal to, and more than 1 indicates synergy, additivity, and antagonism, respectively.

**Table 2:** Combination indices and affected fractions with RITA (5 µM) and DXM (1 µM) combination on primary MM samples.

Sample	Combination indices (CI)			Affected fraction (FA)
	ED50	ED75	ED90	
1	0.75	0.64	0.76	0.73
2	0.73	0.66	0.75	0.67
3	0.60	0.52	0.49	0.61

Notes; A CI of less than, equal to, and more than 1 indicates synergy, additivity, and antagonism, respectively.

## Discussion

In this study, we demonstrated that RITA induces a potent activation of JNK signaling in MM cells. GEP by microarray identified a significant number of genes associated with stress responses leading to apoptosis. Consistent with the up-regulation of c-Jun as observed by microarray studies, we found that RITA-induces phosphorylation of c-Jun in MM cells in a time and dose-dependent manner which causes activation of p53 and cell death. These results suggest the activation of JNK signaling in MM cells upon stimulation by RITA. Activation of JNK by hgal9 (a recombinant protease resistant galectin-9), or plinabulin (a novel vascular disrupting agent), or perifosine (an Akt inhibitor) has previously been reported in MM cells [23], [24], [33]. Accumulating evidence has demonstrated that during apoptotic signaling, activity of both of p53 and c-Jun, can be modulated through posttranslational modifications by JNK cascade [14], [15], [34]. Stabilization and activation of the p53 by JNK signaling has been described in p53 null mouse fibroblast [14], [15]. However, the functional linkage between activation of p53 and JNK signaling has not been elucidated in MM cells induced by p53 reactivating agents such as RITA.

**Figure 8:**
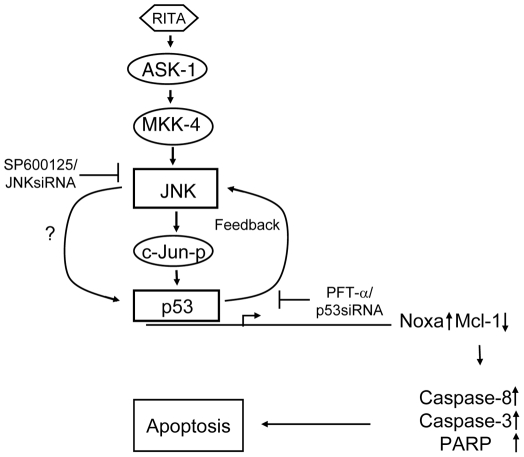
Schematic model for the mechanism of JNK mediated activation of the p53 pathway in RITA-induced MM cells. RITA induces the activation of p53 through c-Jun/JNK signaling pathway by inducing phosphorylation of ASK1-MKK-4 and c-Jun which in turn induces apoptosis by up-regulation of p53 and Noxa; downregulation of Mcl-1, and cleavage of caspase-8, caspase-3 and PARP. Pharmacological (SP600125) or genetic inhibition (JNK siRNA) of activation of c-Jun inhibits RITA-induced activation of p53 and attenuate apoptosis induction by RITA. In addition, treatment of MM cells with p53-transcriptional inhibitor, PFT-α or p53 siRNA abrogates RITA-induced activation of c-Jun and induction of apoptosis. These results suggest the presence of a positive feedback loop between p53 and JNK. Depending on the cellular context, p53 can be directly activated by JNK, although the mechanism is not clear.

Here we provide the first line of evidence that the activation of JNK has a crucial role for efficient induction of apoptosis by pharmacologically activated p53. Off note, the activation of JNK signaling in MM cells was found to be selective for RITA as compared to other nongenotoxic (nutlin) or genotoxic drugs (etoposide). In addition, the JNK activation by RITA appears to be more effective in MM cells in comparison to other tumor cell types. Moreover, we found that induction of p53 is independent of activation of JNK signaling, since RITA induces phosphorylation of c-Jun in cells where p53 was mutated or null. The inhibition of p53 activation upon silencing of JNK suggests that induction of p53 signaling occurs downstream of JNK which is in contrast to the previous studies where JNK activation was described as a downstream event of p53 activation associated with activation of EGR1 [26] and p73 [35]. Another important aspect of our study is that inhibition of activation of p53 transcriptional targets by PFT-α or p53 siRNA resulted in inhibition of phosphorylation of c-Jun. These results indicate the establishment of a positive feedback loop between p53 and JNK (Figure 8) potentiating the apoptosis induction by RITA.

We have demonstrated that activation of JNK is playing an apoptotic role in MM cells induced by RITA, which is consistent with a previous observation showing the requirement of JNK activation JNK for the stabilization of p53 and enhancement of p53 trans-activation by abrogating MDM2 association in p53 null fibroblast [15]. However, depending on the cellular context, c-Jun may play a survival role. These opposing effects have previously been reported for c-Jun and β-catenin, a key component of the Wnt signaling pathway [35] as well as for p53 mediated JNK activation [36]. Activation of JNK in these studies was described as only a downstream event of p53 and inhibition of endogenous JNK activity resulted in an increase of apoptosis in response to nocodazole treatment of human colon carcinoma cells harboring wild type p53 in the latter studies [36].

Based on our results we suggest a schematic model illustrating a novel mechanism of p53-dependent JNK mediated induction of apoptosis by RITA (Figure 8). Stimulation of MM cells by RITA results in activation of JNK through JNK cascade and phosphorylation of c-Jun, which induces p53 accumulation. Activated p53 in turn may enhance JNK signaling through a positive feedback loop between p53 and JNK. JNK activation has previously been shown to phosphorylate p53 (Ser 15) at its N-terminal activation loop [37], [38]. We observed activation of JNK in the absence of phosphorylation of p53 in RITA-induced MM cells (data not shown). Therefore, further study will be required to understand whether JNK can directly activate p53 in MM cells (Figure 8). Based on our data which showed activation of JNK through induction of phosphorylation of JNK upstream kinases, it is unlikely that activation of JNK is mediated by direct interaction of RITA with JNK. Nevertheless, future identification of specific biding target(s) for RITA will improve our understanding on its mechanisms of action and provides a rationale approach for the development of more potent form of RITA for induction of p53-mediated apoptosis.

Although we have provided strong evidence that activation of JNK signaling plays a major role in activation of p53 pathway in MM cells, we can not completely rule out the other pathways leading to p53 activation and subsequent apoptosis of MM cells. Therefore, we also studied the association of other possible pathways in the apoptosis of MM cells induced by RITA as listed in Table S2. We examined modulations of several stress response genes such as up-regulation of ATF3, ATF4, DDIT3, and down-regulation of XBP1 indicative of the unfolded protein response (UPR) including the PERK-eIF2a-CHOP branch of the UPR. Although we found the alterations of these UPR-related genes at mRNA level by qRT-PCR, we could not confirm those changes at the protein level by Western blot analysis (data not shown). However, our data demonstrating a significant inhibition of p53 activation and attenuation of apoptosis upon blockage of JNK activation suggest that JNK signaling is the major pathway in RITA-induced apoptosis of MM cells. These results are consistent with an earlier study in human prostate cancer cells where inhibition of JNK activation strongly reduced p53 induction and almost completely suppressed 2-ME-induced apoptosis [39].

Our results broaden the understanding of the novel role of c-Jun/JNK as an apoptotic regulator in RITA-induced apoptosis of MM cells with functional p53. To our knowledge this is the first report describing that induction of p53-mediated apoptosis by small molecule such as RITA is due to its ability to activate JNK. The present findings could have implications for the design of novel approaches to the treatment of multiple myeloma and possibly other hematopoietic malignancies. Preclinical studies have demonstrated the efficacy of RITA in leukemia [40] as well as in myeloma [9]. In addition, evidence has recently been presented indicating that RITA may potentiate the cytotoxic effects of several novel signal transduction modulators, including MEK inhibitors [41] and 17-AAG [42]. We have previously reported synergistic cytotoxic response of RITA in combination with nutlin [9]. Here, we have demonstrated that RITA potentiate the anti-myeloma activity of DXM in both MM cell lines and patient samples. Caspase-dependent activation of JNK and p38 MAPK by DXM has previously been reported in eosinophil. Treatment of eosinophil with antisense oligonucleotide of JNK1/2 resulted in inhibition of activation of c-Jun [31]. To further examine the significance of JNK activation in RITA-mediated apoptosis we combined RITA with another JNK activator CDDO [32] and examined their cytotoxic effect in MM cells. Similar to the results obtained in combination with DXM, the combination of RITA plus CDDO displayed a synergistic cytotoxic effect in both H929 and MM.1S cells (Figure S3). Taken together, these results suggest that RITA potentiate the anti-myeloma activity of the drugs which can activate JNK and the combination of RITA plus DXM may overcome drug resistance in MM cells.

Our new observations improve understanding of the mechanisms of anti-myeloma activity of RITA and thus may facilitate translation of these findings into the clinic to improve patient outcome in MM. These findings open an approach for the development of anti-myeloma drug(s) with a broader spectrum.

## Supporting Information

Figure S1 **Networks of p53-regulated genes in MM.1S cell lines.** Ingenuity pathways analysis software (Ingenuity Systems) was used to analyze the identified genes (n = 85). The network representing proteins involved in the biologic functions of cancer and the cell cycle is shown. Ingenuity pathway analysis network 1 (A) and 2 (B) depicting relationships among up- and down-regulated genes in MM.1S cells upon RITA treatment. The genes written in bold letters with a shaded node were identified by microarray analysis, and the other genes were those related to the regulated genes based on the network analysis. The intensity of a node color indicates the degree of up-regulation (red). Nodes are displayed using various shapes that represent the functional class of the gene product. Edges are displayed with various labels that describe the nature of relationship between the nodes: ___ represents direct relationship; - - - -represents indirect relationship; →represents acts on.
(TIF)

Figure S2 **Synergistic activity of RITA and DXM in H929 cells (A) and primary MM samples (B).** Isobologram and FA-CI plots were produced by CalcuSyn software. The leftward shift of the dose response curve indicates synergistic response. Values below the threshold line represent synergistic combination. The data shown in Figure B are representative of the 3 separate experiments performed in different primary samples.
(TIF)

Figure S3 **Combination of RITA and the known JNK activator CDDO induces synergistic cytotoxicity in MM cells.** (A) MM.1S and H929 cells were treated with different concentrations of CDDO (0, 0.25, 0.5, 1.0 µM). After 48 hrs, the viability of the cells was measured by MTT assay. (B) MM.1S and H929 cells were simultaneously treated with low doses (0.25–1.0 µM) of RITA, and 0.5 µM CDDO for 48 hrs, followed by assessment for cell viability using MTT assays. Data are mean ±SD of triplicate samples. *p<0.05 versus the samples treated with RITA or CDDO alone (Student's *t* test).
(TIF)

Table S1 **Nucleotide sequences of the primers used for qRT-PCR.**
(DOC)

Table S2 **Molecules associated with RITA-induced signaling pathways in MM.1S cells.**
(DOC)
