# Supplementary figures and images for: Targeting p53 via JNK Pathway: A Novel Role of RITA for Apoptotic Signaling in Multiple Myeloma
Source: PLoS One. 2012 Jan 20;7(1):e30215. doi: 10.1371/journal.pone.0030215 (PMC3262803; doi:10.1371/journal.pone.0030215)

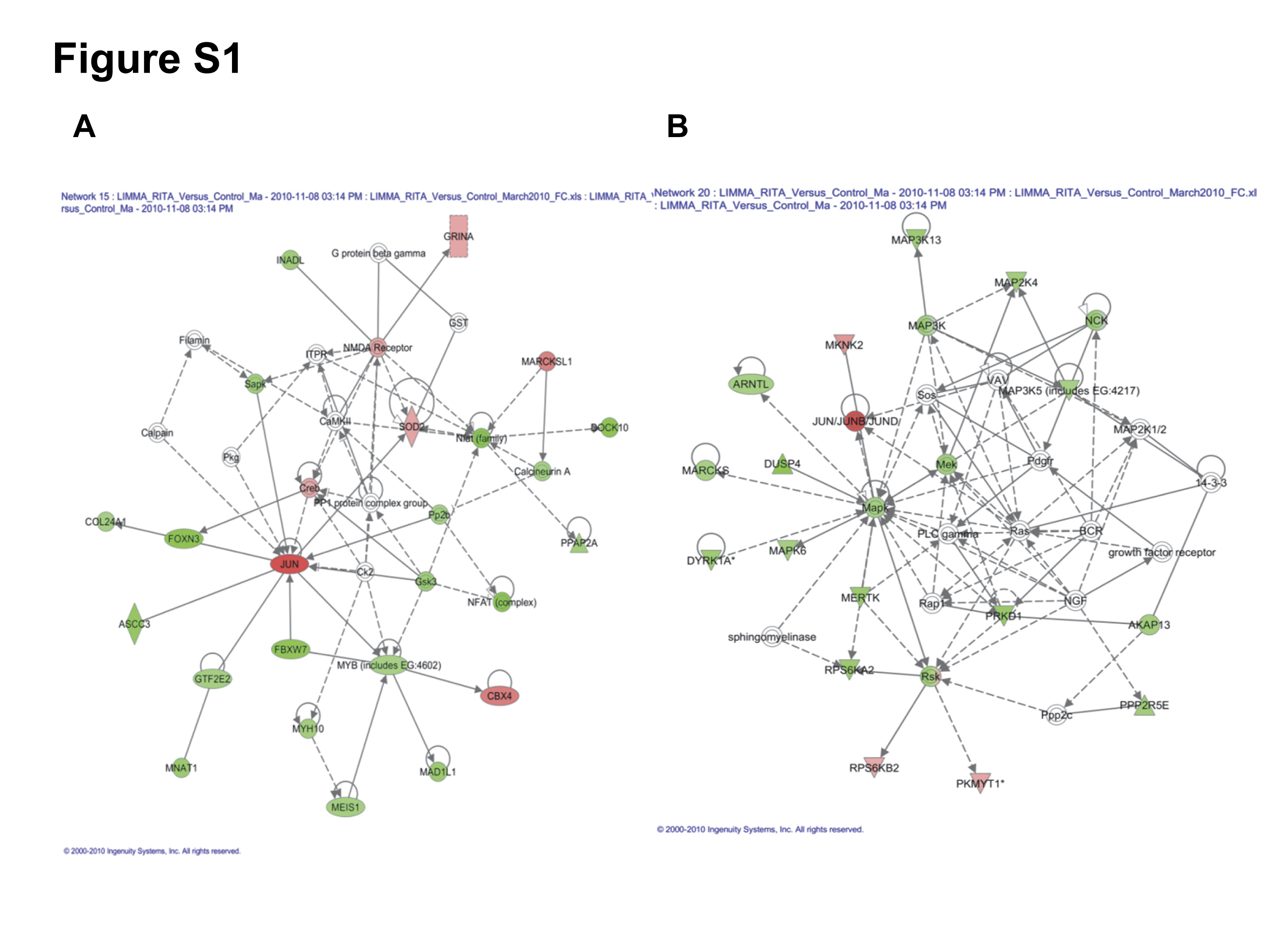

Supplement: Figure S1 — Networks of p53-regulated genes in MM.1S cell lines. Ingenuity pathways analysis software (Ingenuity Systems) was used to analyze the identified genes (n = 85). The network representing proteins involved in the biologic functions of cancer and the cell cycle is shown. Ingenuity pathway analysis network 1 (A) and 2 (B) depicting relationships among up- and down-regulated genes in MM.1S cells upon RITA treatment. The genes written in bold letters with a shaded node were identified by microarray analysis, and the other genes were those related to the regulated genes based on the network analysis. The intensity of a node color indicates the degree of up-regulation (red). Nodes are displayed using various shapes that represent the functional class of the gene product. Edges are displayed with various labels that describe the nature of relationship between the nodes: ___ represents direct relationship; - - - -represents indirect relationship; →represents acts on. (TIF) [file pone.0030215.s001.tif]

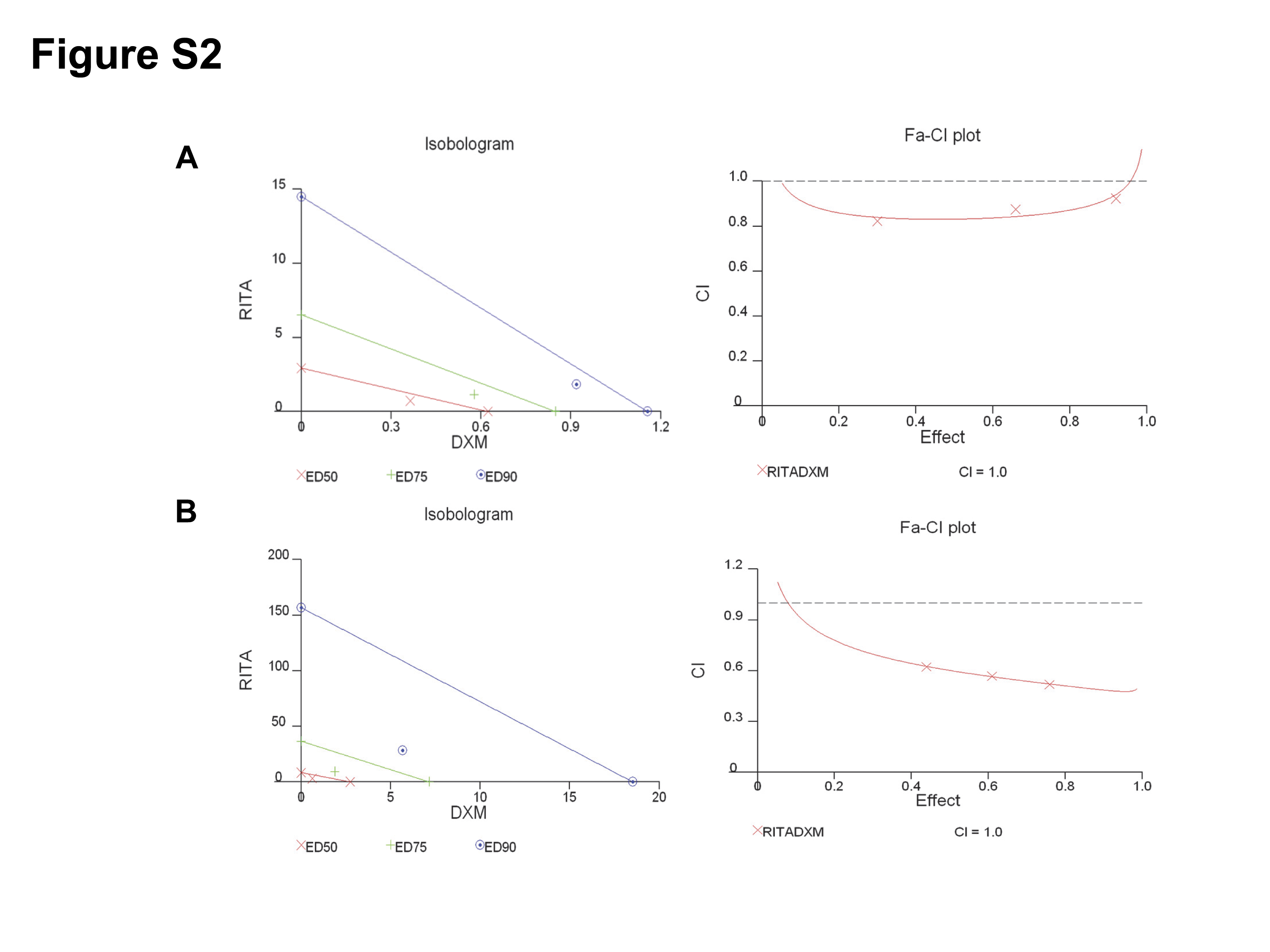

Supplement: Figure S2 — Synergistic activity of RITA and DXM in H929 cells (A) and primary MM samples (B). Isobologram and FA-CI plots were produced by CalcuSyn software. The leftward shift of the dose response curve indicates synergistic response. Values below the threshold line represent synergistic combination. The data shown in Figure B are representative of the 3 separate experiments performed in different primary samples. (TIF) [file pone.0030215.s002.tif]

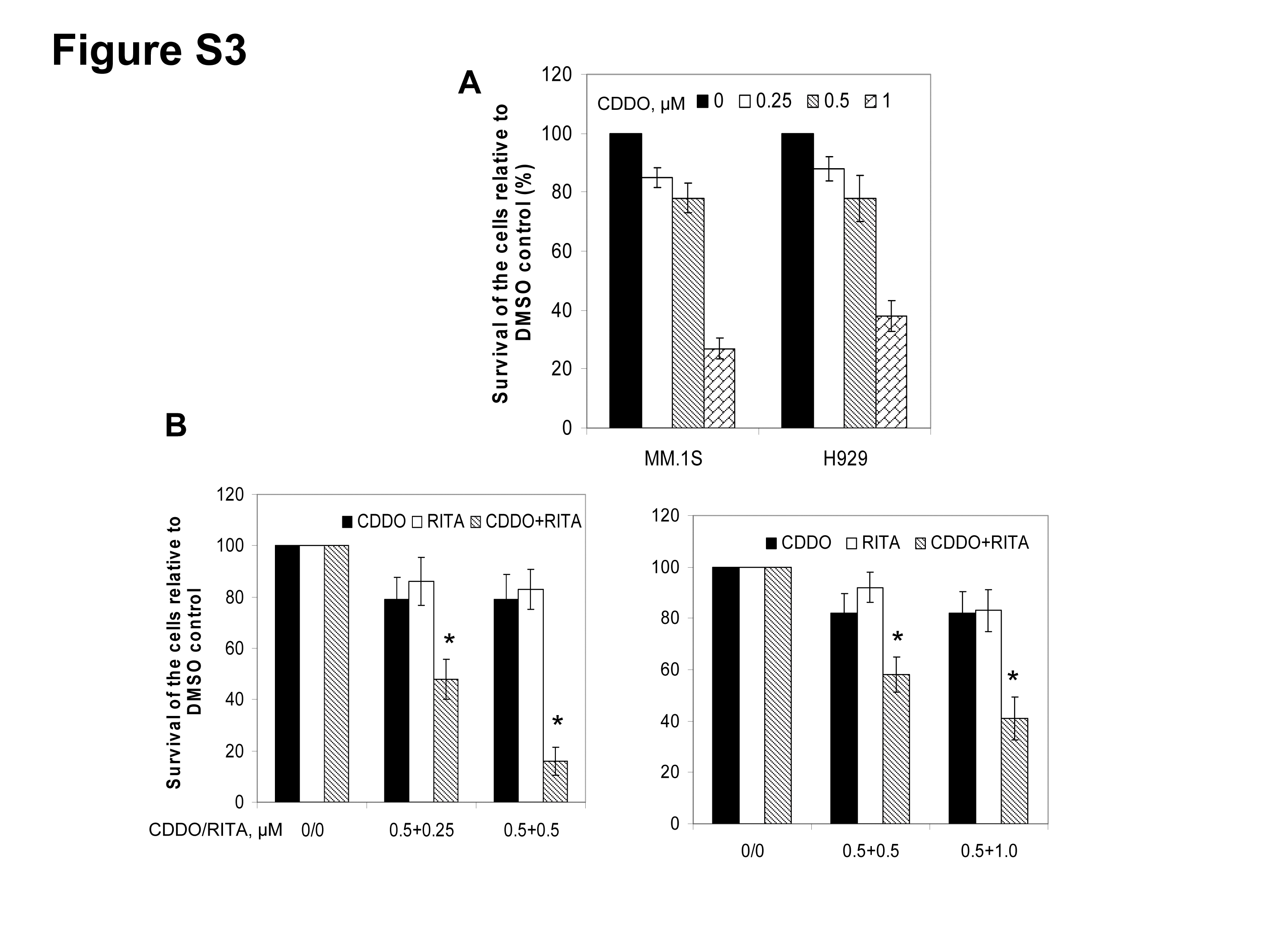

Supplement: Figure S3 — Combination of RITA and the known JNK activator CDDO induces synergistic cytotoxicity in MM cells. (A) MM.1S and H929 cells were treated with different concentrations of CDDO (0, 0.25, 0.5, 1.0 µM). After 48 hrs, the viability of the cells was measured by MTT assay. (B) MM.1S and H929 cells were simultaneously treated with low doses (0.25–1.0 µM) of RITA, and 0.5 µM CDDO for 48 hrs, followed by assessment for cell viability using MTT assays. Data are mean ±SD of triplicate samples. *p<0.05 versus the samples treated with RITA or CDDO alone (Student's t test). (TIF) [file pone.0030215.s003.tif]
