# Supplementary material for: Targeting p53 via JNK Pathway: A Novel Role of RITA for Apoptotic Signaling in Multiple Myeloma
Source: PLoS One. 2012 Jan 20;7(1):e30215. doi: 10.1371/journal.pone.0030215 (PMC3262803; doi:10.1371/journal.pone.0030215)
Supplement: Table S1 — Nucleotide sequences of the primers used for qRT-PCR. (DOC) [file pone.0030215.s004.doc]

Table S1. Nucleotide sequences of the primers used for qRT-PCR

| Gene name | Primer (5′ to 3′) |
| --- | --- |
| ATF3 | Forward: AGA AGG AAC ATT GCA GAG CTA AG  Reverse: GGATTC TAG AGGTAC ACA GGA AG |
| ATF4 | Forward: TCTCATTCAGGCTTCTCACGGCAT  Reverse: AAGCTCATTTCGGTCATGTTGCGG |
| DDIT3 | Forward: ATGGCAGCTGAGTCATTGCCTTTC  Reverse: AGAAGCAGGGTCAAGAGTGGTGAA |
| DDIT4 | Forward: TACAGCTCGGAACAGCTGCTCATT  Reverse: TTCCAACCACAGGAATCAGTCCCT |
| JUN | Forward: TCG ACATGG AGT CCC AGG A  Reverse: GGC GAT TCT CTC CAG CTT CC |
| FOS | Forward: CGAGCCCTTTGATGACTTCCT  Reverse: GGAGCGGGCTGTCTCAGA |
| GADD45A | Forward: AAG GAT GGA TAA GGT GGG G  Reverse: CTG GAT CAG GGT GAA GTG G |
| MYC | Forward: AAT GAA AAG GCC CCC AAG GTA GTT ATC C  Reverse: GTC GTT TCC GCA ACA AGT CCT CTT C |
| BCL2 | Forward: CTG CAC CTG ACG CCCTTC ACC  Reverse: CAC ATG ACC CCA CCG AACTCA AAG A |
| GAPDH | Forward: ACA TCG CTC AGA CAC CAT G  Reverse: TGT AGT TGA GGT CAA TGA AGG G |

Note. MM.1s cells were treated with 1.0 µm RITA for 6 hrs and mRNA levels for a panel of p53-responsive transcripts were then analyzed by qRTPCR using the listed primers.
