# Supplementary material for: Targeting p53 via JNK Pathway: A Novel Role of RITA for Apoptotic Signaling in Multiple Myeloma
Source: PLoS One. 2012 Jan 20;7(1):e30215. doi: 10.1371/journal.pone.0030215 (PMC3262803; doi:10.1371/journal.pone.0030215)
Supplement: Table S2 — Molecules associated with RITA-induced signaling pathways in MM.1S cells. (DOC) [file pone.0030215.s005.doc]

**Table S2. Molecules associated with RITA-induced signaling pathways in MM.1S cells**

| **Pathways** | **Molecules** |
| --- | --- |
| Molecular Mechanisms of Cancer | MAP2K4, TCF4, PIK3R1, ARHGEF7, ABL1, MAP3K5, NFKB1, BCL2, TGFBR2, MYC, JUN, RHOB, RHOD, PRKCE, ARHGEF3, GSK3B, PMAIP1, NFKBIB, RASA1, PRKD1, PRKCA, CREBBP, CDK6, RHOJ, AURKA, FOS, CBL, FOXO1, GAB1, CDKN1A, PIK3CB, LEF1 |
| PI3K/AKT Signaling | PIK3R1, MAP3K5, NFKB1, PTEN, BCL2, FOXO1, GAB1, PPP2R3A, CDKN1A, FOXO3, RPS6KB2, PIK3CB, GSK3B, PPP2R5E, NFKBIB |
| Endoplasmic Reticulum Stress Pathway | XBP1, ATF4, ATF6, MAP3K5, EIF2AK3, XBP1, ATF4, ATF6, MAP3K5, EIF2AK3 |
| 14-3-3-mediated Signaling | MAP2K4, PIK3R1, PLCL2, MAP3K5, FOS, JUN, CBL, FOXO1, PRKCE, PIK3CB, GSK3B, PRKD1, PRKCA |
| PTEN Signaling | CBL, FOXO1, PIK3R1, CDKN1A, FOXO3, RPS6KB2, PIK3CB, GSK3B, NFKB1, PTEN, BCL2 |
| ATM Signaling | MAP2K4, JUN, GADD45A, CDKN1A, ABL1, CCNB2, ATF4, TLK2 |
| p53 Signaling | JUN, GADD45A, STAG1, PIK3R1, CDKN1A, PIK3CB, GSK3B, LRDD, PMAIP1, PTEN, BCL2 |
| Cell Cycle: G1/S Checkpoint Regulation | MYC, HDAC4, CDKN1A, CUL1, CDK6, ABL1, HDAC9, GSK3B |
| mTOR Signaling | DDIT4, PRKAB1, PIK3R1, RHOJ, RHOB, RHOD, PPP2R3A, RPS6KB2, PRKCE, PIK3CB, RPS6KA2, PPP2R5E, PRKD1, PRKCA |
| EGF Signaling | MAP2K4, FOS, JUN, PIK3R1, PIK3CB, RASA1, PRKCA |
| PPARα/RXRα Activation | MAP2K4, PRKAB1, NCOA6, CREBBP, CKAP5, ACVR1, PLCL2, NFKB1, NCOA3, TGFBR2, JUN, PRIC285, NFKBIB, PPARGC1A, PRKCA |
| Cell Cycle: G2/M DNA Damage Checkpoint Regulation | GADD45A, CDKN1A, CUL1, TOP2A, CCNB2, PKMYT1 |
| SAPK/JNK Signaling | MAP2K4, JUN, GADD45A, GAB1, NFATC3, PIK3R1, MAP3K13, DUSP4, PIK3CB, MAP3K5 |

|  |  |
| --- | --- |

**Table S2. Continued.**

| **Pathways** | **Molecules** |
| --- | --- |
| IGF-1 Signaling | FOS, JUN, IGF1, FOXO1, PIK3R1, FOXO3, IGF1R, RPS6KB2, PIK3CB, RASA1 |
| Role of Wnt/GSK-3β Signaling in the Pathogenesis of Influenza | TCF4, CSNK1G1, CSNK1G3, NCOA1, LEF1, GSK3B, WNT5B, NCOA3 |
| Wnt/β-catenin Signaling | TCF4, CSNK1G1, CSNK1G3, SOX12, CREBBP, ACVR1, MYC, TGFBR2, PPP2R3A, TLE4, LEF1, GSK3B, PPP2R5E, WNT5B |
| TNFR1 Signaling | MAP2K4, FOS, JUN, CRADD, NFKBIB, NFKB1 |
| Myc Mediated Apoptosis Signaling | MAP2K4, MYC, IGF1, PIK3R1, IGF1R, PIK3CB, BCL2 |
| p38 MAPK Signaling | MAP2K4, TGFBR2, MYC, DDIT3, ATF4, MEF2C, MKNK2, MAP3K5, EEF2K |
| ERK/MAPK Signaling | ETS1, PIK3R1, MKNK2, ELF1, MYC, FOS, ELF2, PPP2R3A, PRKCE, ATF4, DUSP4, PIK3CB, PPP2R5E, PRKCA |
| Death Receptor Signaling | MAP2K4, CRADD, MAP3K5, NFKBIB, NFKB1, BCL2 |
| PPAR Signaling | FOS, JUN, CREBBP, NCOA1, NFKBIB, NFKB1, PPARGC1A |
| Apoptosis Signaling | MAP2K4, PRKCE, MAP3K5, NFKBIB, NFKB1, PRKCA, BCL2 |
| NFκB Signaling | TLR4, PIK3R1, CREBBP, PIK3CB, MALT1, GSK3B, NFKBIB, NFKB1 |
| Inhibition of Angiogenesis by TSP1 | TGFBR2, JUN |
| Chemokine Signaling | FOS, JUN, CCL5, PRKCA |
| Notch Signaling | RFNG, NUMB |
| JAK/Stat Signaling | PIK3R1, CDKN1A, PIK3CB |

Note. MM.1s cells were treated with 1.0 µm RITA for 6 hrs and differential expression of the genes between RITA-treated and DMSO-treated samples were examined by gene expression analysis. Differentially expressed genes and their association with the signaling pathways were further analysed by IPA.
